# Impact of Metabolic Syndrome and It's Components on Prognosis in Patients With Cardiovascular Diseases: A Meta-Analysis

**DOI:** 10.3389/fcvm.2021.704145

**Published:** 2021-07-15

**Authors:** Xiao Li, Yajing Zhai, Jiaguo Zhao, Hairong He, Yuanjie Li, Yue Liu, Aozi Feng, Li Li, Tao Huang, Anding Xu, Jun Lyu

**Affiliations:** ^1^Department of Clinical Research, The First Affiliated Hospital of Jinan University, Guangzhou, China; ^2^Department of Clinical Medicine, Qinghai Institute of Health Sciences, Xining, China; ^3^Department of Pharmacy, The First Affiliated Hospital of Xi'an Medical University, Xi'an, China; ^4^Department of Orthopaedic Surgery, Tianjin Hospital, Tianjin, China; ^5^Clinical Research Center, The First Affiliated Hospital of Xi'an Jiaotong University, Xi'an, China; ^6^Department of Human Anatomy, Histology and Embryology, School of Basic Medical Sciences, Xi'an Jiaotong University Health Science Center, Xi'an, China; ^7^Xiyuan Hospital of China Academy of Chinese Medicinal Sciences, Beijing, China; ^8^Department of Neurology, First Affiliated Hospital of Jinan University, Guangzhou, China

**Keywords:** cardiovascular disease, metabolic syndrome, all-cause death, prognosis, meta-analysis

## Abstract

**Background:** Patients with metabolic syndrome (MetS) have a higher risk of developing cardiovascular diseases (CVD). However, controversy exists about the impact of MetS on the prognosis of patients with CVD.

**Methods:** Pubmed, Cochrane library, and EMBASE databases were searched. Cohort Studies and randomized controlled trials *post hoc* analyses that evaluated the impact of MetS on prognosis in patients (≥18 years) with CVD were included. Relative risk (RR), hazard rate (HR) and 95% confidence intervals (CIs) were calculated for each individual study by random-effect model. Subgroup analysis and meta-regression analysis was performed to explore the heterogeneity.

**Results:** 55 studies with 16,2450 patients were included. Compared to patients without MetS, the MetS was associated with higher all-cause death [RR, 1.220, 95% CI (1.103 to 1.349), *P*, 0.000], CV death [RR, 1.360, 95% CI (1.152 to 1.606), *P*, 0.000], Myocardial Infarction [RR, 1.460, 95% CI (1.242 to 1.716), *P*, 0.000], stroke [RR, 1.435, 95% CI (1.131 to 1.820), *P*, 0.000]. Lower high-density lipoproteins (40/50) significantly increased the risk of all-cause death and CV death. Elevated fasting plasma glucose (FPG) (>100 mg/dl) was associated with an increased risk of all-cause death, while a higher body mass index (BMI>25 kg/m^2^) was related to a reduced risk of all-cause death.

**Conclusions:** MetS increased the risk of cardiovascular-related adverse events among patients with CVD. For MetS components, there was an increased risk in people with low HDL-C and FPG>100 mg/dl. Positive measures should be implemented timely for patients with CVD after the diagnosis of MetS, strengthen the prevention and treatment of hyperglycemia and hyperlipidemia.

## Introduction

Cardiovascular disease (CVD) has attracted worldwide attention and accounts for 46.2% of deaths from non-communicable diseases (1). CVD is one of the main causes of premature death and disability. Metabolic syndrome (MetS), including dysglycemia, obesity (especially central obesity), high blood pressure, low high-density lipoprotein cholesterol (HDL-C), and elevated triglyceride levels, is a complex of risk factors for type 2 diabetes and CVD (2). Patients with MetS have a higher risk of developing CVD compared with those without MetS in the next 5–10 years, and the long-term risk is even higher (3). The National Cholesterol Education Program (NCEP) Adult Treatment Panel (ATP) III criteria also considered MetS as the second major target for CVD prevention (4).

The prevalence of MetS is higher in patients with CVD than in patients without MetS. The prevalence of MetS in hospitalized patients with acute myocardial infarction (AMI) is 46%, similar to that of the acute coronary syndrome (43.4%) (5); This finding indicates that MetS is associated with CVD. Boulon et al. (6) suggested that despite active management, patients with MetS have a higher long-term risk of cardiovascular events (6). However, Selcuk et al. (7) suggested that the main determinant of long-term prognosis of AMI is heart failure rather than metabolic disorder (7). But some researchers suggested that MetS does not increase the mortality among patients with CVD (8). Therefore, controversy exists about the impact of MetS on patients with CVD.

MetS is a disease associated with multiple factors, and the main diagnostic indicators (components) include blood pressure, overweight and obesity, HDL-C, and fasting blood glucose (9, 10). Most studies have focused on the overall effect of MetS on the prognosis of CVD. However, whether a correlation exists between each component and prognosis and which factor is more important have not been elucidated. Considering these inconsistencies, we performed a meta-analysis of cohort studies and RCT *post-hoc* analysis from CVD patients to evaluate associations between different definitions of MetS and the risk of all cause death, CV death and cardiovascular events.

## Methods

The study was registered with PROSPERO (CRD42021147609), and reported in accordance with the PRISMA statement (11).

### Inclusion Criteria

#### Eligible Studies

(1) Influencing factors and study types: Studies that evaluated the influence of MetS and its components on patients with CVD were included. We included cohort and randomized controlled trials *post hoc* analyses and excluded single-group observational studies. (2) Types of patients: Patients with CVD were aged ≥18. (3) Outcomes: Primary outcomes were all-cause death, cardiovascular (CV) death, incidence of MI and stroke. Secondary outcomes were TVR, heart failure, cardiac arrest, angina pectoris, cardiogenic shock. All-cause death of high TG, low HDL-C, high BP, FPG>100 mg/dl, BMI>25kg/m^2^, high WC. CV death of high TG, low HDL-C, high BP, FPG>100 mg/dl, BMI>25kg/m^2^.

The definition of cardiovascular disease in this meta-analysis was history (comorbidity) of cardiovascular or cardiac disease. Hypertension/Cardiovascular Infections/Cardiovascular Abnormalities/Pregnancy Cardiovascular Complications/cardiomyopathy in specific terms was excluded because these diseases often overlap and potentially result in overestimation of cases.

#### Exclusion Criteria

(1) Studies that had incomplete or unavailable original data. (2) The diagnostic criteria for MetS were not specified. (3) Repeated published data. (4) Studies that evaluated the relationship between MetS and congenital heart disease.

### Data Sources and Searches

We searched Pubmed, EMBASE, and Cochrane library from inception to October 18, 2020. The following subject and keywords were used in search: “cardiovascular disease,” “cardiovascular event,” “cardiocerebrovascular disease,” “cerebrovascular disease,” “cerebrovascular disorder,” “cerebrovascular attack,” “stroke,” “cerebral infarction,” “coronary artery disease,” “coronary heart disease,” “ischemic heart disease,” “myocardial infarction;” “metabolic syndrome,” “metabolic syndrome x,” “Metabolic X Syndrome;” “Randomized controlled trial,” “RCT,” “Clinical Trials, Randomized,” “Cohort Studies,” “Follow-Up Studies,” “Longitudinal Studies,” “Prospective Studies,” and “Retrospective Studies”. Supplementary Table 1 presents the search strategy. No date, language, or other restriction were incorporated into the searches. Two researchers (XL and YJZ) performed the data search.

### Study Selection

Endnote X9 was used to manage and screen the literature. Title, abstract, and full texts were selected based on inclusion/exclusion criteria. We designed a standardized form to extract data including study characteristics, diagnostic criteria, characteristics of the study population, risk of bias, and outcome measures.

### Risk of Bias Analysis

We used the Newcastle–Ottawa Scale (NOS) to assess the quality of the cohort studies (12). To be specific, studies with scores >7 were treated as high quality, 4–6 as medium quality, and below 4 as low quality (13). Cochrane Collaboration's tool for assessing the risk of bias was applied to determine the quality of the included RCT *post-hoc* studies (12, 14). Two researchers (X Li, YJ Zhai) independently screened and extracted the data, and a third researcher (J Lyu) resolved any disagreements. Quality evaluation results are reported in Supplementary Table 2.

The diagnostic criteria for MetS vary among different regions and institutions, but the majority of them included central obesity, hypertension, low HDL-C, and high TG and fasting blood glucose (FBG) levels. Other diagnostic criteria also included dyslipidemia, chronic mild inflammation, endothelial dysfunction, insulin resistance, increased oxidative stress. The diagnostic criteria used in the included studies were NCEP2001 criteria (9), NCEP2005 criteria (4), and The International Diabetes Federation (IDF) criteria (10) (details reported in Supplementary Table 3). For specific diagnostic criteria, we compared the above criteria and divide into subgroups based on the comparison results.

### Statistical Analysis

Statistical analysis was performed using STATA 13 and R software. For dichotomous outcomes (all-cause death, CV-death, the incidence of MI, stroke, TVR, heart failure, cardiac arrest, angina pectoris, and cardiogenic shock), relative risk (RR) and 95% confidence intervals (CIs) were calculated for each individual study. For the impact of MetS components on patients with CVD (all-cause death and CV death), hazard rate (HR) and 95% confidence intervals (CIs) were determined for each study. The heterogeneity across studies was examined using the Chi-square test and I-square statistics. The results were pooled by the D-L random-effect model due to the large statistical heterogeneity among the studies.

To explore the sources of clinical heterogeneity and methodological heterogeneity, we performed subgroup analysis based on the following: (1) diagnostic criteria, studies were divided into four subgroups (NCEP2001, NCEP2005, IDF and “others”) and (2) study type, studies were divided into three subgroups (prospective cohort study, retrospective cohort study, and RCT *post-hoc* study). Meta-regression analysis of three covariates (follow-up time, male proportion, and patient age) was performed to explore the size and source of heterogeneity.

Effect measures [risk ratio (RR) vs. odds ratio (OR) vs. risk difference (RD)] and statistical models (D-L random-effects model vs. M-H fix-effects model) were used to examine the robustness of the results. We evaluated publication bias by Begg's tests and drew contour-enhanced funnel plots to assess whether the asymmetry of the funnel plots was caused by publication bias or other biases.

## Results

### Overview of the Characteristics of the Studies

**Figure 1:**
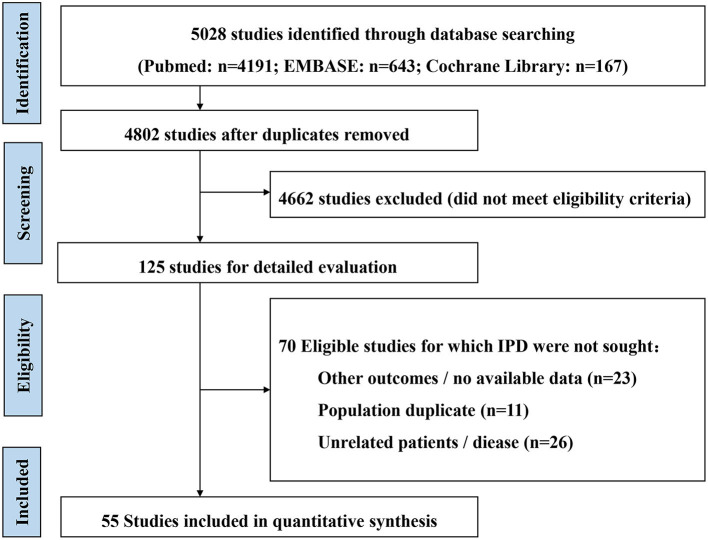
Flow chat of study selection.

A total of 5,028 unique records were identified from the literature search. After excluding 226 duplicate articles, 125 studies were initially included by reading the title and abstract. Fifty-five studies were finally included after further reading the full text, including six RCT *post-hoc* studies (15–20) and 49 cohort studies (3, 5–8, 21–64) (Figure 1).

### Study Characteristics

A total of 162,450 patients from 25 countries and regions were included, the sample size for each individual study varies from 57 to 44 548. Forty-one studies (145,390 patients) evaluated the risk of all-cause death among patients with CVD and MetS. Twenty-one studies with 95,049 patients reported CV death, 23 studies with 77,618 patients reported the incidence of MI, and 11 studies with 59,770 patients reported the incidence of stroke.

Twenty-six studies adopted NCEP-ATPIII (2005) criteria, 21 studies mainly adopted NCEP-ATPIII (2001) criteria, and 7 studies adopted IDF (2005). Baseline characteristics are listed in Table 1. Risk of bias was assessed in all of the 55 studies (Supplementary Table 2). The cohort studies comprised 16 medium-quality studies, and 33 high-quality studies. For RCT *post-hoc* studies, the risk of bias was deemed low in 2 studies and moderate in 4 studies.

**Table 1:** Characteristics of included studies.

**No**.	**Author**	**Year**	**Country**	**Study design**	**Follow-up (Years)**	**Sample**	**Male (%)**	**Age (years)**	**Endpoints**	**Definition of MetS**
1	Anderson	2004	America	Retrospective cohort study	2.80 ± 2.30	2,035	76.00	65 ± 11	②③	NCEP2001
2	Marroquin	2004	America	Prospective cohort study	3.50 (2.80–4.70)	284	0.00	58 ± 12	①②③④⑦	NCEP2001
3	Rana	2005	Netherlands	Prospective cohort study	at least 0.75	901	NA	62 ± 11	②⑤③	NCEP2005
4	Saely	2005	Australia	Prospective cohort study	2.30 ± 0.40	750	67.90	62.6 ± 10.4	②③④⑤	NCEP2001
5	Schwartz	2005	America	RCT *post-hoc* analysis	0.33	3,038	65.00	65 ± 12	①③⑥⑧⑩	NCEP2001
6	Zeller	2005	France	Prospective cohort study	6.90	633	75.00	66.2	①③④⑨	NCEP2001
7	Aguilar	2006	America, New England, Canada	RCT *post-hoc* analysis	3.10	3,319	81.70	62 ± 11	①③⑤⑧	NCEP2001
8	Boulon	2006	France	Prospective cohort study	1.60	480	82.20	61.6 ± 13	①④⑤⑦	NCEP2001,IDF
9	Briand	2006	Canada	Retrospective cohort study	2.30 ± 1.10	105	62.00	69 ± 12	①	NCEP2001
10	Hu	2006	China	Retrospective cohort study	2.30 ± 1.00	2,596	77.70	60.3 ± 10.3	②③④⑤⑦	IDF
11	Kasai	2006	Janpan	Retrospective cohort study	12.00 ± 3.60	748	87.00	59 ± 10	①②⑩	NCEP2001
12	Nigam	2006	Canada	Retrospective cohort study	12.60 ± 5.10	24,958	75.60	52.9 ± 9.3	①③④⑦	NCEP2001
13	Ovbiagele	2006	America	RCT *post-hoc* analysis	1.80	476	61.60	63 ± 11.4	②③④⑩	NCEP2001
14	Espinola-Klein	2007	Germany	Retrospective cohort study	6.70	811	75.10	62.7 ± 9.3	②③④	NCEP2005
15	Hajer	2007	Netherlands	Prospective cohort study	2.80 (0.10–7.50)	2,060	78.00	59.6 ± 10.3	②④	NCEP2001
16	Nakatani	2007	Janpan	Prospective cohort study	2.00	3,858	76.00	64.7 ± 11.4	②③	NCEP2001
17	Canibus	2007	Italy	Prospective cohort study	1.00	148	79.70	61 ± 11	②⑤	NCEP2001
18	Espinola-Klein	2007	Germany	Prospective cohort study	6.10 (0.70–7.70)	1,263	74.40	61.6 ± 10.1	②	NCEP2005
19	Iturry-Yamamoto	2009	Brazil	Prospective cohort study	1.00	159	71.70	60.7 ± 10.6	②③⑤	NCEP2005
20	Kasai	2009	Janpan	Retrospective cohort study	11.40 ± 2.90	1,836	85.10	59.2 ± 9.0	①②④⑩	NCEP2005
21	Protack	2009	America	Retrospective cohort study	4.50	921	64.00	71 ± 10	②③④	Custom
22	Selcuk	2009	Turkey	Prospective cohort study	2.30 (1.20–3.50)	188	82.40	56.9 ± 11.6	②③⑤	NCEP2005
23	Solymoss	2009	Canada	Retrospective cohort study	12.60 ± 3.40	1,080	73.40	58.1 ± 9.8	①②③④⑧	NCEP2005
24	Suwaidi	2010	Bahrain, Kuwait, Qatar, Oman, United Arab Emirates, and Yemen	Prospective cohort study	0.50	6,701	75.70	56.4 ± 12.2	①③④⑦	NCEP2005
25	Lee	2010	Korea	Prospective cohort study	1.00	1,990	73.00	63.4 ± 12.6	①②③⑤	NCEP2005
26	Miller	2010	Mexico	Prospective cohort study	/	971	70.00	62.3 ± 11.5	①⑤⑦	NCEP2005
27	Petersen	2010	America	Prospective cohort study	5.00	5,744	64.60	62(53–71)	①③④⑩	NCEP2005
28	Van Kuijk	2010	Netherlands	Retrospective cohort study	6.00 (2.00–9.00)	2,069	81.40	/	②③⑤⑩	NCEP2001
29	Hoshida	2011	Janpan	Prospective cohort study	1.00	1,173	72.50	67	①②③④⑤	NCEP2005
30	Hu	2011	China	Prospective cohort study	2.95	1,224	71.70	60 ± 10	②③	IDF
31	Kalahasti	2011	America	Retrospective cohort study	1.00	2,362	73.00	64	①③⑤⑩	Custom
32	Maron	2011	America	RCT *post-hoc* analysis	4.60 (2.50–7.00)	2,248	85.10	62.1 ± 9.9	①③⑤⑦⑩	NCEP2005
33	Capoulade	2012	Canada	Prospective cohort study	3.40 ± 1.30	243	62.00	57 ± 13	②	NCEP2001
34	Marso	2012	Netherlands	Prospective cohort study	3.00	673	75.80	58.2(50.1–70.8)	②③⑧	NCEP2001
35	Mi	2012	China	Prospective cohort study	1.00	701	64.80	61.4 ± 11.7	①④⑩	IDF
36	Arnold	2013	America	Prospective cohort study	1.00	1,129	66.00	59.7 ± 11.6	①	NCEP2001
37	Balti	2013	France	Prospective cohort study	5.00	57	56.00	61.9 ± 12.9	①	NCEP2005
38	Hossain	2014	Bangladesh	Prospective cohort study	1.00	210	70.00	53.2 ± 12	①④⑥⑨	NCEP2005
39	Mehta	2014	New England, Canada, America, Australian	RCT *post-hoc* analysis	1.00	9,406	68.40	68(60–75)	①	NCEP2005
40	Mornar	2014	Croatia	Prospective cohort study	1.00	250	/	/	②③	NCEP2005
41	Udell	2014	America	Prospective cohort study	4.00	44,548	64.60	68.7 ± 10.4	①②③④	NCEP2005
42	Won	2014	Korea	Prospective cohort study	3.00	963	75.60	62 ± 12	①②③⑩	NCEP2005
43	Ao	2015	China	Retrospective cohort study	5.00	1,238	84.40	59.5 ± 9	①③④	NCEP2005
44	Arbel	2015	Russia	Prospective cohort study	4.40 ± 1.90	3,525	72.00	66 ± 22	①	NCEP2005
45	Fan	2015	China	Retrospective cohort study	2.30	997	69.90	64.29 ± 13.13	⑦①	Custom
46	Perrone-Filardi	2015	Italy	Substudy of RCT	3.00	6,648	78.20	67.2 ± 10.6	①②⑩	IDF
47	Simao	2015	Brazil	Retrospective cohort study	1.00	148	56.80	69.5(55–81.5)	①	NCEP2005
48	Chen	2016	China	Prospective cohort study	4.90	3,351	63.00	64 ± 2.4	①②⑩	NCEP2005
49	Fang	2016	China	Prospective cohort study	3.40	1,087	51.20	65.1 ± 8.9	②③④	Custom
50	La Carrubba	2016	Italy	Prospective cohort study	1.80	1,920	56.30	60(50–69)	②③④⑤	IDF
51	Tadaki	2016	Janpan	Retrospective cohort study	3.20 ± 1.10	4,566	68.00	68.8 ± 1.4	①③⑤⑧	NCEP2001
52	Bhagat 2017	2017	Indian	Prospective cohort study	2.00	358	74.90	56.19 ± 11.56	①⑧⑨	NCEP2005
53	Lovic	2018	Serbia	Prospective cohort study	4.00	507	77.71	58.57 ± 11.30	①②③④⑤	AHA-NHLBI(NCEP2005), NCEP2001 and IDF
54	Vest	2018	USA	Prospective cohort study	5.10 (2.20–8.20)	1,953	74.00	55 (48–63)	①	NCEP2001
55	Polovina	2018	Serbia	Prospective cohort study	5.00	843	61.40	62.5 ± 12.2	②③⑩	NCEP2005

*① All-cause death, ② CV death, ③ MI, ④ Stroke, ⑤ TVR, ⑥ Cardiac arrest, ⑦ HF, ⑧ Angina pectoris, ⑨ Cardiogenic shock, ⑩ MetS components*.

### Meta-Analysis Results

#### All-Cause Death and CV Death

**Figure 2:**
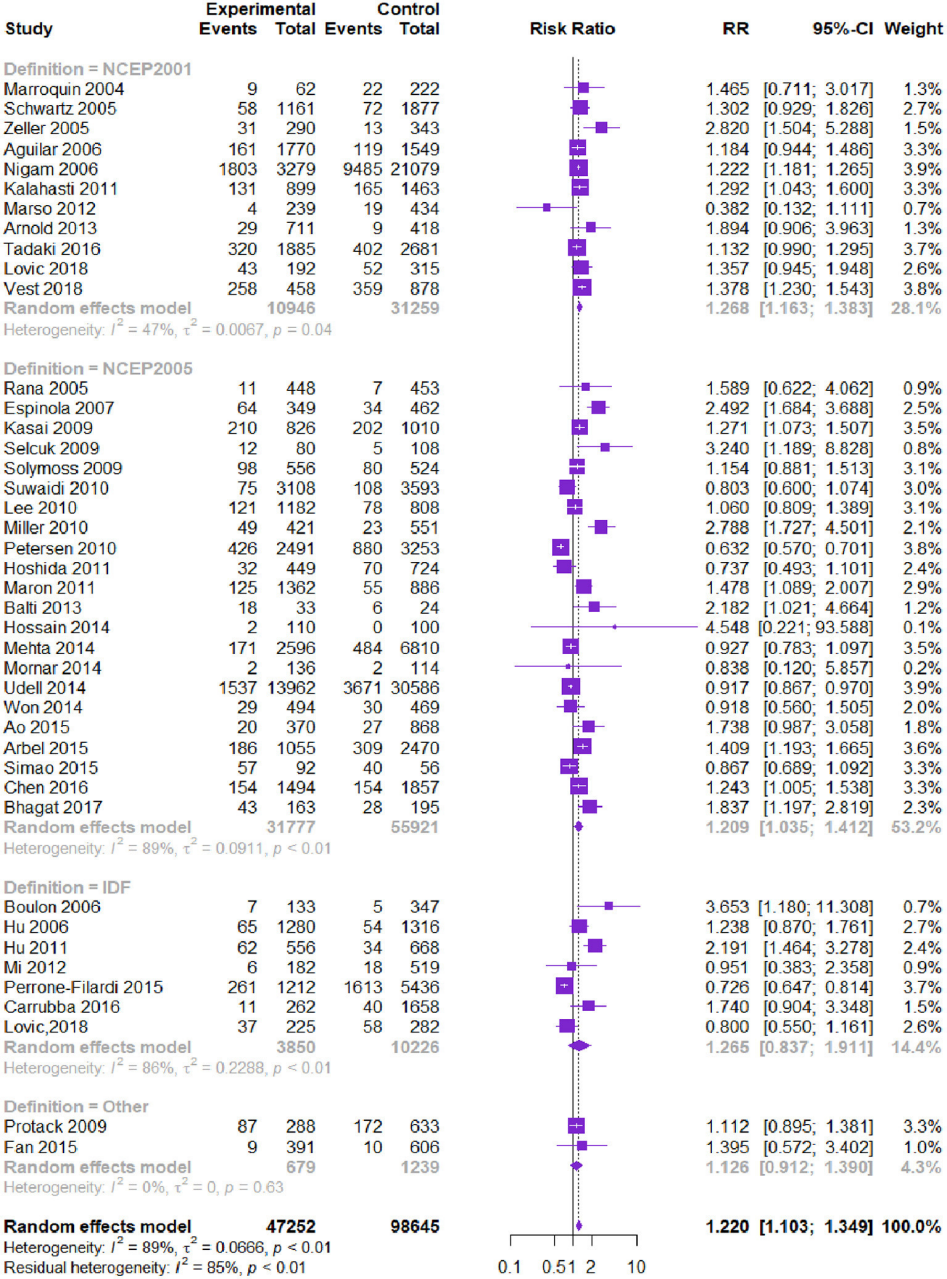
Meta-analysis of the risk of all-cause death in patients with CVD and MetS compared with that of patient without MetS.

Forty-one studies (145,897 patients) reported all-cause death. MetS was associated with higher all-cause death [RR = 1.220, 95% CI (1.103, 1.349), *P* = 0.000] according to the heterogeneity test I2 = 89% (Table 2, Figure 2). Subgroup analysis showed that among different diagnostic criteria of MetS, the results from NCEP-ATPIII (2001) and NCEP-ATPIII (2005) subgroups were consistent with the overall result (Table 3). Among different study types, the cohort study subgroup was in the same direction with the overall results. No statistically significant difference was found in the RCT *post-hoc* studies. Diagnostic criteria and study type were the factors that affected heterogeneity. Meta-regression showed that the follow-up time and male proportion were not the sources of heterogeneity (*P* > 0.05), and age only explained 1.6% of the heterogeneity (*P* = 0.022). The Begg's test result showed bias (*P* = 0.012), and the contour-enhanced funnel plots showed that the bias may be due to other reasons rather than publication bias.

**Table 2:** The main results of meta-analysis.

**Outcome**	**Number of studies**	**Number of volunteers**	**Random effect model RR (95%CI)**	***P***	***I^**2**^*(%)**	**Begg's Test *P***
**Primary outcomes**						
All-cause death	41	145,897	1.22 (1.10–1.35)	<0.01	89	0.01
CV death	21	94,542	1.36 (1.15–1.61)	<0.01	87	0.02
MI	23	77,125	1.46 (1.24–1.72)	<0.01	72	0.13
Stroke	11	60,297	1.44 (1.13–1.82)	<0.01	75	0.01
**Secondary outcomes**						
TVR	13	17,072	1.241 (1.06–1.45)	<0.01	81	0.16
Angina pectoris	3	5,147	1.28 (0.97–1.69)	0.03	71.5	–
Heart failure	8	12,369	1.50 (1.12–2.01)	<0.01	88.5	–
Cardiac arrest	4	4,171	1.46 (0.88–2.43)	0.52	0.0	–
Cardiogenic shock	3	7,309	1.28 (0.97–1.69)	0.03	71.5	–

**Table 3:** The results of subgroup analysis based on diagnostic criteria.

**Outcome**	**Subgroup**	**Number of studies**	**RR (95%CI)**	***P***	***I^**2**^*(%)**
All-cause Death	NCEP2001	11	1.27 (1.16–1.38)	<0.01	47
	NCEP2005	22	1.21 (1.04–1.41)	0.02	89
	IDF	7	1.27 (0.84–1.91)	0.19	86
	Other	2	1.13 (0.91–1.39)	0.27	0
CV Death	NCEP2001	5	1.67 (1.15–2.43)	0.01	68
	NCEP2005	11	1.45 (1.13–1.86)	<0.01	83
	IDF	4	1.02 (0.58–1,81)	0.93	80
	Other	/	/	/	/
MI	NCEP2001	7	1.57 (1.04–2,36)	0.03	81
	NCEP2005	12	1.18 (1.08–1.28)	<0.01	7
	IDF	3	1.58 (0.96–2.59)	0.07	16
	Other	2	2.24 (0.91–5.51)	0.08	91
Stroke	NCEP2001	3	1.77 (1.25–2.51)	<0.01	0
	NCEP2005	4	1.21 (0.89–1.64)	0.22	81
	IDF	3	1.79 (1.04–3.11)	0.04	0
	Other	2	1.45 (1.05–2.02)	0.03	25
TVR	NCEP2001	4	1.34 (0.91–1.96)	0.14	74
	NCEP2005	6	1.22 (1.08–1.37)	<0.01	0
	IDF	3	1.33 (0.84–2.09)	0.22	86

**Figure 3:**
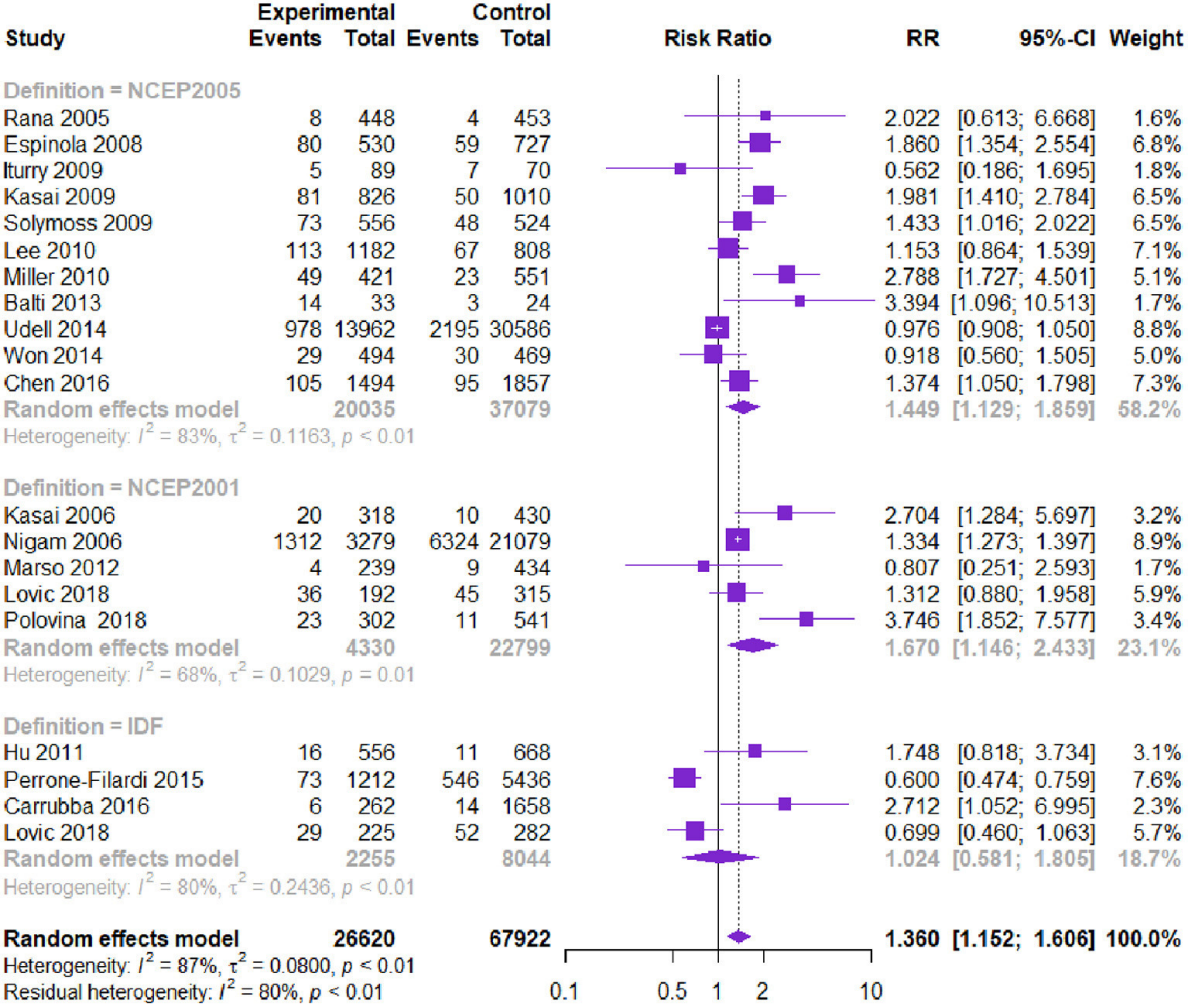
Meta-analysis of the risk of CV death in patients with CVD and MetS compared with that of patient without MetS.

Twenty-one studies with 94,542 patients reported CV-related death. The MetS group had higher CV death than the non-MetS group [RR = 1.360, 95% CI (1.152, 1.606), *P* = 0.000] according to the heterogeneity test I^2^=87.0% (Table 2, Figure 3). Subgroup analysis showed that among different diagnostic criteria of MetS, NCEP-ATPIII (2001) and NCEP-ATPIII (2005) subgroups were consistent with the overall result (Table 3). Among different study types, the subgroups were consistent with the overall results. Diagnostic criteria affected the heterogeneity. Meta-regression showed that follow-up time, age, and male proportion were not the source of heterogeneity (*P* > 0.05). The Begg's test and the contour-enhanced funnel plots showed that bias may be caused by publication bias and other reasons.

#### Risk of MI and Stroke

**Figure 4:**
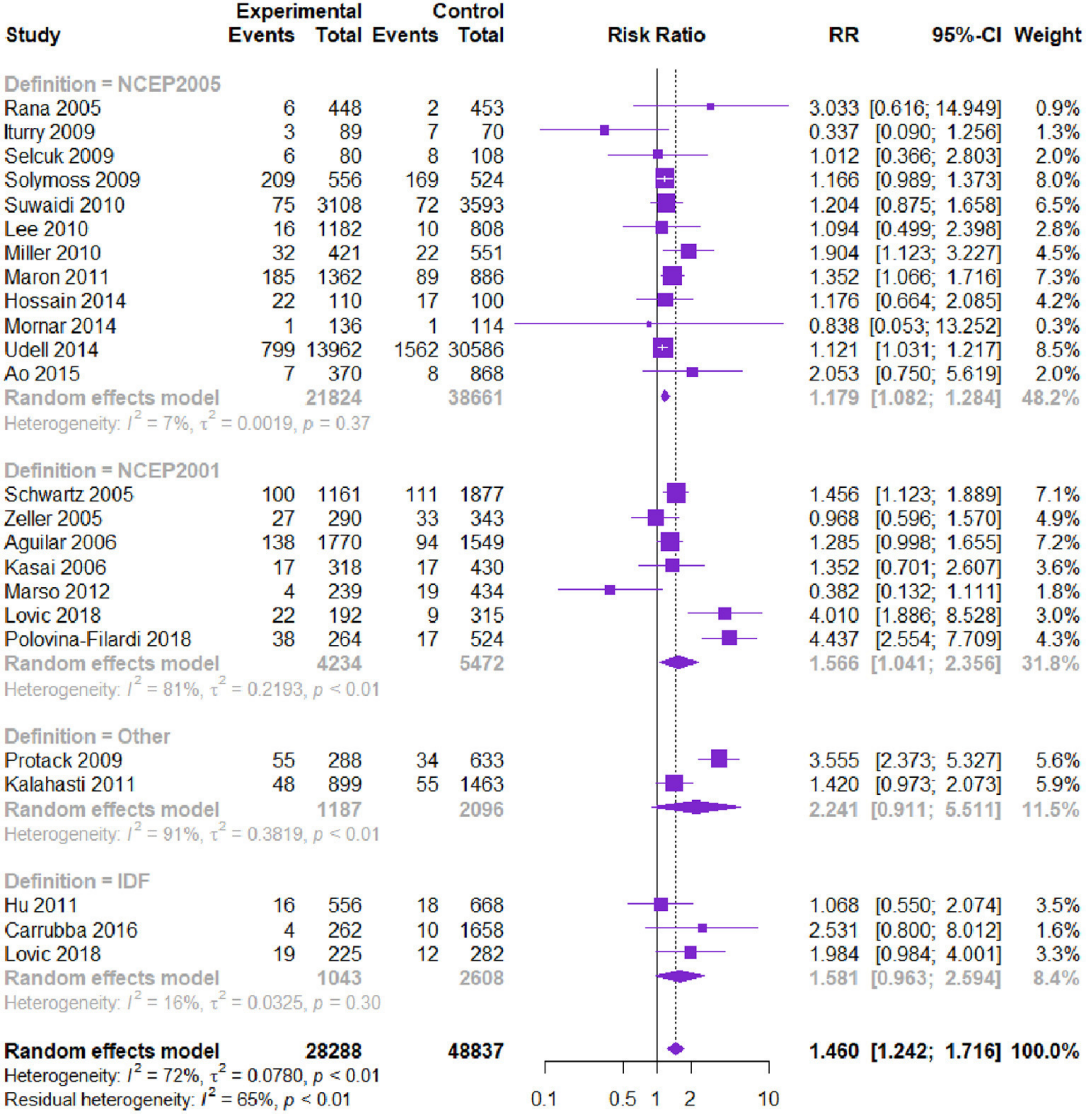
Meta-analysis of the risk of MI in patients with CVD and MetS compared with that of patients without MetS.

Twenty-three studies with 77,125 patients reported the risk of MI. Patients with CVD and MetS had a higher risk of MI [RR = 1.460, 95% CI (1.242, 1.716), *P* = 0.000] according to the heterogeneity test I^2^ = 72% (Table 2, Figure 4). Subgroup analysis showed that among the diagnostic criteria of MetS, the results of NCEP-ATPIII (2001) and NCEP-ATPIII (2005) were consistent with the overall results (Table 3). Other subgroups had no statistically significant difference. Among the study types, the subgroup results were in the same direction as the overall results. Meta-regression showed that follow-up time, age, and male proportion were not the source of heterogeneity (*P* > 0.05). The Begg's test and the contour-enhanced funnel plots reported no publication bias (*P* = 0.125).

**Figure 5:**
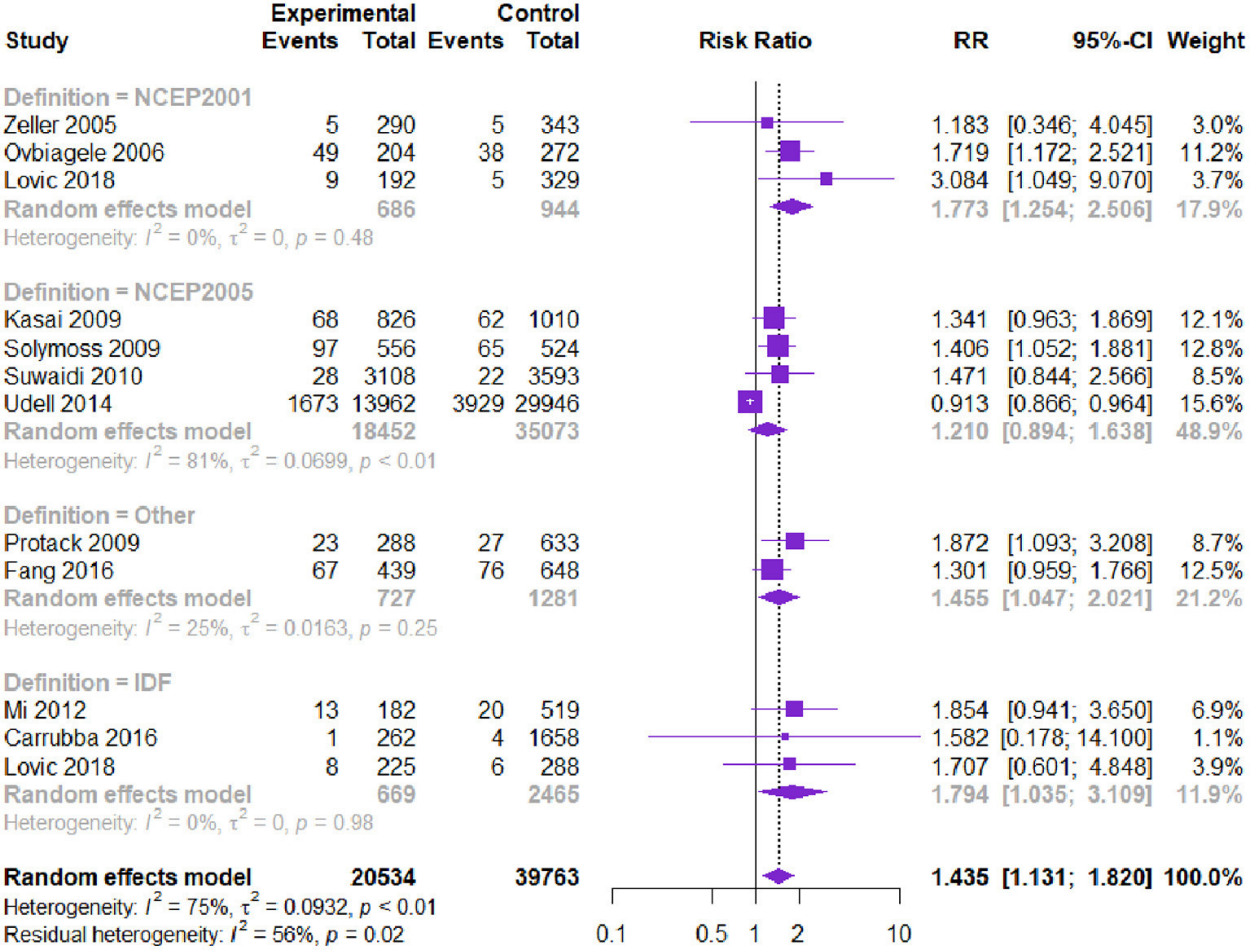
Meta-analysis of the risk of stroke in patients with CVD and MetS compared with that of patients without MetS.

Eleven studies with 60,297 patients reported the risk of stroke. Patients with CVD and MetS had a higher risk of stroke [RR = 1.435, 95% CI (1.131, 1.820), *P* = 0.000] according to the heterogeneity test I^2^ = 75% (Table 3, Figure 5). The Begg's test and the contour-enhanced funnel plots showed that the bias may be caused by other reasons rather than publication bias.

#### Other Adverse Cardiovascular Indicators

The results of the TVR (13 studies) reported that patients with CVD and MetS had a higher risk to develop TVR [RR = 1.241, 95% CI (1.063, 1.448), *P* = 0.000]. Subgroup analysis showed that diagnostic criteria and study type explained the partial heterogeneity. The risk of heart failure was evaluated in eight studies. Patients with CVD and MetS were more likely to have heart failure [RR = 1.497, 95% CI (1.116, 2.007), *P* = 0.000]. Subgroup analysis showed that diagnostic criteria partly explained the heterogeneity (Table 2).

Other indicators include risk of cardiac arrest (4 studies), angina pectoris (3 studies), and cardiogenic shock (3 studies). We found no statistically significant difference in the risk of cardiac arrest [RR = 1.457, 95% CI (0.875, 2.429), *P* = 0.518], angina pectoris [RR = 1.280, 95% CI (0.967, 1.694), *P* = 0.030], and cardiogenic shock [RR = 0.923, 95% CI (0.752 1.132), *P* = 0.764].

#### Impact of MetS Component

Among MetS components, low HDL (40/50) was significantly associated with increased risks of all-cause death and CV death. Elevated FPG (>100 mg/dl) was significantly associated with an increased risk of all-cause death, whereas body mass index (BMI) > 25 kg/m^2^ was related to a reduced risk of all-cause death (Table 4).

**Table 4:** The results of metabolic syndrome's components.

**Components**	**Outcomes [HR (95%CI)]**				
	**Number of studies**	**All-cause death**	***I^**2**^* (%)**	**Number of studies**	**CV death**	***I^**2**^* (%)**
High TG	9	0.97 (0.93–1.01)	68	2	0.89 (0.77–1.03)	0
Low HDL-C	8	1.17(1.09–1.26)	56	2	1.39 (1.00–1.94)	74
High BP	9	0.98(0.94–1.01)	71	2	0.82 (0.58–1.18)	69
FPG>100 mg/dl	11	1.29 (1.23,1.35)	61	2	1.24 (0.96–1.60)	53
BMI>25 kg/m^2^	5	0.88(0.79, 0,97)	89		/	
High WC	2	0.91(0.49–1.69)	36		/	

### Sensitivity Analysis

We examined the robustness of our results. The sensitivity analysis of the effect measures showed that the OR increased the effect size and did not change the direction of the results, except for angina pectoris. The RD did not change the direction of the results. The sensitivity analysis of the statistical models did not change the direction of the results. Hence, the results of this meta-analysis were robust.

## Discussion

### Summary of Main Results

Fifty-five studies with 162,450 patients from 25 countries or regions were included. Most studies defined MetS using NCEP2001, NCEP2005, and IDF criteria, and other works adopted specific diagnostic criteria. Our results suggested that patients with CVD and MetS had an increased risk of all-cause death, CV-related death, MI, stroke, TVR, and heart failure. In the analysis of MS components, BMI>25 kg/m^2^ was negatively correlated with the prognosis of patients with CVD. Dyslipidemia and abnormal glucose metabolism were the main risk factors for the prognosis of CVD. Different spectrum within patients with cardiovascular diseases may be the sources of heterogeneity.

### Potential Biases in the Review Process

MetS and its components are a complex of risk factors for CVD and diabetes (21). Ford (65) reported that the population attributable fractions for CVD, diabetes, and all-cause death among patients with MS were 12–17%, 30–52%, and 6–7%, respectively (65). However, for patients with CVD, whether MetS and its components is associated with the risk of CV events remains controversial.

Obesity is an independent risk factor for hypertension, CVD, and diabetes (66). Given the known association between obesity and CVD, the adverse consequences of obesity may persist after the onset of CVD. However, previous studies suggest a contradictory U-shaped relationship between obesity and CVD-related death; hence, overweight and mild obesity are related to lower short-term and long-term mortality (67–69) based on the concept of “Obesity paradox” or “reverse epidemiology” (66). Although the setting of obesity indicators was involved in different MetS diagnostic criteria, the core of the diagnosis was consistent. In NCEP-ATP III (2001) and NCEP-ATP III (2005) criteria, obesity is one of the five elements and is not a necessary condition; however, in IDF (2005), obesity is the first prerequisite. Interestingly, our result discovered that the diagnosis of MetS under different standards has a distinct prognosis of CVD. The result of the subgroup analysis of all-cause mortality and cardiovascular mortality as two core factors demonstrated that IDF (2005) standards were consistently different from the final result. However, under the standards of NCEP-ATP III (2001) and NCEP-ATP III (2005) who didn't consider obesity as a necessary condition, MetS is a significant risk factor of prognosis. Hence, we need to reconsider which diagnostic criteria can predict the prognosis of MetS among patients with CVD more accurately. The heterogeneity in this study may be associated with the proportion of obese patients included.

AHA/NHLBI 2009 diagnostic criteria were not adopted in all of the included studies, which may be related to the fact that the indicators and numerical intervals of abdominal obesity were not clearly given in the criteria. BMI was used in most of the studies as a proxy for waist circumference, but the cutoffs for the inclusion criteria in each study were different. This phenomenon may be related to two factors: (1) BMI is easier to obtain than waist circumference, and (2) BMI can be effectively docked with the WHO's definition of obesity. However, existing evidence suggests that MetS might be caused by excessive central obesity (70). Therefore, in future research on MetS, we suggest that BMI and waist circumference data should be collected at the same time for strict implementation of MetS diagnostic criteria.

### Impact of Follow-Up Time on Results

The span of follow-up time included was very large, ranging from 0.33 years to 12.6 years. A 32-year prospective cohort study of male residents without MI or stroke in the community showed that the CV-related mortality curves among patients with MetS varied at 10–15 years of follow-up (70). The findings of Kasai et al. (26) and Nigam et al. (46) show that MetS and its components had a significantly positive association with all-cause death of patients with CVD during 4–5 years of follow-up (20, 26). However, the impact of MetS on patients with CVD might be underestimated in these studies.

### MS Components of Study

In this study, the potential influences of the five components of MetS [TG, HDL, BP, FPG, BMI/Waist circumference (WC)] on CVD prognosis was analyzed. We found that abnormal blood glucose and lipid metabolism are important factors that could lead to poor prognosis of CVD. As such, these factors should be considered as intervention targets for predicting the prognosis of patients with CVD. BMI was negatively correlated, which was manifested as the obesity paradox. Waist circumference was included in only two studies with relatively small sample sizes and conducted among Chinese patients only. Further studies are needed to explore the rationality, applicability, and the risk prognosis of BMI and waist circumference.

Prediabetes is an intermediate metabolic state between normoglycemia and diabetes, includes impaired glucose tolerance and impaired fasting glucose (71). Compared with NCEP-ATP III (2001) criteria, the NCEP-ATP III (2005) reduced the fasting plasma glucose from 6.1 mmol/L to 5.6 mmol/L. Our results showed that the two diagnostic criteria had the same contribution in predicting the prognosis of patients with CVD. The results of Huang 2016 also found that prediabetes with impaired fasting glucose or impaired glucose tolerance is associated with an increased risk of composite cardiovascular events, coronary heart disease, stroke, and all-cause mortality (71). Our findings indirectly supported the modification of the American ADA guidelines to reduce the standard of pre-diabetes from 6.1 mmol/L (72) to 5.6 mmol/L (73). In response to this result, lifestyle intervention is the fundamental management approach for prediabetes (73, 74).

### Limitations

The span of follow-up time included was very large, ranging from 0.33 to 12.6 years, most studies were followed up for <5 years, the impact of MetS on patients with CVD in this study might be underestimated. As one of the diagnostic indicators of MetS, WC was only included in two studies, reflected the problems in the implementation of MetS diagnostic criteria and possibly underestimated the impact of central obesity on patients with CVD.

## Conclusions

This meta-analysis was conducted using cohort studies and RCT *post-hoc* studies. MetS was found to be associated with an increased risk of CV-related adverse events among patients with CVD. For MetS components, there was an increased risk in people with low HDL-C and FPG>100 mg/dl. Positive measures should be implemented timely for patients with CVD after the diagnosis of MetS to reduce risk factors and strengthen the prevention and treatment of hyperglycemia and hyperlipidemia. Further studies need to clarify the selection of MetS diagnostic indicators (particularly the BMI or waist circumference).

## Data Availability Statement

The original contributions presented in the study are included in the article/Supplementary Material, further inquiries can be directed to the corresponding authors.

## Author Contributions

JL, AX, XL, and YZ conceived the study. YZ and HH designed the search strategy and XL performed the literature search. JZ and YZ screened studies for eligibility. HH, YLi, YLiu, and AF performed data extraction. XL, YZ, LL, and TH assessed the risk of bias. XL, YZ, HH, and JZ performed data analysis. JL interpreted the data analysis and assessed the certainty of evidence. XL and YZ wrote the first draft of the manuscript and all other authors revised the manuscript. All authors contributed to the article and approved the submitted version.

## Conflict of Interest

The authors declare that the research was conducted in the absence of any commercial or financial relationships that could be construed as a potential conflict of interest.
