# Supplementary material for: Impact of Metabolic Syndrome and It's Components on Prognosis in Patients With Cardiovascular Diseases: A Meta-Analysis
Source: Front Cardiovasc Med. 2021 Jul 15;8:704145. doi: 10.3389/fcvm.2021.704145 (PMC8319572; doi:10.3389/fcvm.2021.704145)
Supplement: Supplementary file 1 [file Table_1.docx]

| **Search number** | **Query** |
| --- | --- |
| 6 | #1 and #2 and #5 |
| 5 | #3 or #4 |
| 4 | (((((("cohort studies"[MeSH Terms]) OR ("cohort studies"[Text Word])) OR ("cohort studies"[Title/Abstract])) OR ("follow up studies"[Text Word])) OR ("follow up studies"[Title/Abstract])) OR ("longitudinal studies"[Text Word])) OR ("longitudinal studies"[Title/Abstract]) |
| 3 | (((((((("randomized controlled trials as topic"[MeSH Terms]) OR ("randomized controlled trials"[Text Word])) OR ("randomized controlled trials"[Title/Abstract])) OR ("rct"[Title/Abstract])) OR ("rct"[Text Word])) OR ("randomized clinical trials"[Text Word])) OR ("randomized clinical trials"[Title/Abstract])) OR ("randomized controlled clinical trials"[Title/Abstract])) OR ("randomized controlled clinical trials"[Text Word]) |
| 2 | ((((((((((((((((((((((("cardiovascular diseases"[MeSH Terms]) OR ("cardiovascular disease"[Title/Abstract])) OR ("cardiovascular event"[Title/Abstract])) OR ("cardiocerebrovascular disease"[Title/Abstract])) OR ("cardiocerebrovascular disease"[Text Word])) OR ("cardiovascular event"[Text Word])) OR ("cardiovascular disease"[Text Word])) OR ("cerebrovascular disease"[Text Word])) OR ("cerebrovascular disease"[Title/Abstract])) OR ("cerebrovascular disorder"[Title/Abstract])) OR ("cerebrovascular disorder"[Text Word])) OR ("cerebrovascular attack"[Text Word])) OR ("cerebrovascular attack"[Title/Abstract])) OR ("stroke"[Title/Abstract])) OR ("stroke"[Text Word])) OR ("cerebral infarction"[Text Word])) ) OR ("cerebral infarction"[Title/Abstract])) OR ("coronary artery disease"[Title/Abstract])) OR ("coronary artery disease"[Text Word])) OR ("coronary heart disease"[Text Word])) OR ("coronary heart disease"[Title/Abstract])) OR ("myocardial infarction"[Title/Abstract])) OR ("myocardial infarction"[Text Word]) |
| 1 | (("metabolic syndrome"[MeSH Terms])) OR ("metabolic syndrome" [Title/Abstract]) |

**Table S1. search strategy**
