# Supplementary material for: Impact of Metabolic Syndrome and It's Components on Prognosis in Patients With Cardiovascular Diseases: A Meta-Analysis
Source: Front Cardiovasc Med. 2021 Jul 15;8:704145. doi: 10.3389/fcvm.2021.704145 (PMC8319572; doi:10.3389/fcvm.2021.704145)
Supplement: Supplementary file 2 [file Table_2.docx]

| No. | study | Random sequence generation | Allocation concealment | Blinding of participants and personnel | Blinding of outcome assessment | Incomplete outcome data | Selective reporting. | Other sources of bias. |
| --- | --- | --- | --- | --- | --- | --- | --- | --- |
| 1 | Schwartz 2015 | Low | Unclear | Low | Low | High | Low | Low |
| 2 | Aguilar 2005 | Unclear | Unclear | Unclear | Unclear | Low | Low | Low |
| 3 | Ovbiagele 2006 | Low | Unclear | Low | Low | Low | Low | Low |
| 4 | Maron 2011 | Unclear | Unclear | Unclear | Unclear | Low | Low | Low |
| 5 | Mehta 2014 | Unclear | Unclear | Low | Low | Unclear | Low | Low |
| 6 | Perrone-Filardi 2015 | Low | Low | Low | Low | Low | Low | Low |

Table S2-1 The risk of bias for RCT post-hoc studies

Table S2-2 the Newcastle–Ottawa Scale (NOS) scores for cohort studies

|  | included studies | Selection | | | | Comparability | | Outcome | | | Score |
| --- | --- | --- | --- | --- | --- | --- | --- | --- | --- | --- | --- |
|  |  | Representativeness of the exposed cohort | Selection of the non- exposed cohort | Ascertainment of exposure | Demonstration that outcome of interest was not present at start of study | Comparability of cohorts on the basis of the design or analysis 'a' | Comparability of cohorts on the basis of the design or analysis 'b' | Assessment of outcome | Was follow-up long enough for outcomes to occer (>5 years) | Adequacy of follow up of cohorts |  |
| 1 | Anderson 2003 | d | a | a | b | Yes | Yes | a | b | d | 5 |
| 2 | Marroquin 2004 | c | a | a | a | No | Yes | c | b | b | 5 |
| 3 | Rana 2005 | d | a | b | a | Yes | Yes | a | b | d | 6 |
| 4 | Saely 2005 | b | a | a | a | Yes | Yes | a | b | d | 7 |
| 5 | Zeller 2005 | b | a | a | a | No | Yes | a | a | b | 8 |
| 6 | Boulon 2006 | b | a | a | a | Yes | Yes | a | b | b | 8 |
| 7 | Brand 2006 | b | a | a | b | Yes | Yes | a | b | d | 5 |
| 8 | Hu 2006 | b | a | a | b | Yes | Yes | a | b | c | 6 |
| 9 | Kasai 2006 | b | a | a | b | Yes | Yes | a | a | a | 8 |
| 10 | Nigam 2005 | b | a | a | b | Yes | Yes | a | a | b | 8 |
| 11 | Espinolar-Klein 2007 | b | a | a | b | Yes | Yes | a | a | b | 8 |
| 12 | Hajer 2007 | b | a | a | a | Yes | Yes | a | b | b | 8 |
| 13 | Nakatani 2007 | b | a | a | a | Yes | Yes | a | b | b | 8 |
| 14 | Canibus 2007 | b | a | a | a | Yes | Yes | a | b | b | 8 |
| 15 | Espinola-Klein 2007 | b | a | a | a | Yes | Yes | a | a | b | 9 |
| 16 | Iturry-Yamamoto 2009 | b | a | a | a | Yes | Yes | a | b | d | 7 |
| 17 | Kasai 2009 | b | a | a | b | Yes | Yes | a | a | a | 8 |
| 18 | Protack 2009 | b | a | a | b | No | No | a | b | a | 5 |
| 19 | Selcuk 2009 | b | a | a | a | Yes | Yes | a | b | a | 8 |
| 20 | Solymoss 2009 | b | a | a | b | Yes | No | a | a | a | 7 |
| 21 | Suwaidi 2010 | a | a | a | a | No | Yes | a | b | a | 7 |
| 22 | Lee 2010 | a | a | a | a | No | Yes | a | b | a | 7 |
| 23 | Miller 2010 | c | a | a | a | No | Yes | a | b | a | 6 |
| 24 | Petersen 2010 | a | a | a | a | No | No | a | a | b | 7 |
| 25 | Van Kuijk 2010 | b | a | a | b | No | Yes | a | a | a | 7 |
| 26 | Hoshida 2011 | b | a | a | a | No | No | a | b | a | 6 |
| 27 | Hu 2011 | b | a | a | a | Yes | Yes | a | b | b | 8 |
| 28 | Kalahasti 2011 | b | a | a | b | No | Yes | a | b | a | 6 |
| 29 | Capoulade 2012 | b | a | a | a | Yes | Yes | a | b | a | 8 |
| 30 | Marso 2012 | a | a | a | a | No | Yes | a | b | a | 7 |
| 31 | Mi 2012 | b | a | a | a | Yes | Yes | a | b | a | 8 |
| 32 | Arnold 2013 | b | a | a | a | No | Yes | a | b | b | 7 |
| 33 | Balti 2013 | b | a | a | a | Yes | Yes | a | a | a | 9 |
| 34 | Hossain 2014 | b | a | a | a | Yes | No | a | b | a | 7 |
| 35 | Mornar 2014 | b | a | a | a | Yes | Yes | a | b | a | 8 |
| 36 | Udell 2014 | a | a | a | a | No | No | a | b | a | 6 |
| 37 | Won 2014 | b | a | a | a | Yes | Yes | a | b | b | 8 |
| 38 | Ao 2015 | b | a | a | b | No | No | a | a | a | 6 |
| 39 | Arbel 2015 | b | a | a | a | Yes | Yes | a | b | a | 8 |
| 40 | Fan 2015 | b | a | a | b | No | No | a | b | a | 5 |
| 41 | Simao 2015 | b | a | a | b | Yes | Yes | a | b | a | 7 |
| 42 | Chen 2016 | b | a | a | a | No | No | a | b | a | 6 |
| 43 | Fang 2016 | a | a | a | a | Yes | Yes | a | b | a | 8 |
| 44 | La Carrubba 2016 | b | a | a | a | Yes | Yes | a | b | a | 8 |
| 45 | Tadaki 2016 | a | a | a | b | No | Yes | a | b | b | 6 |
| 46 | Bhagat 2017 | c | a | a | a | Yes | No | a | b | a | 6 |
| 47 | Lovic 2018 | c | a | a | a | No | Yes | a | b | d | 5 |
| 48 | Vest 2018 | a | a | a | a | Yes | Yes | a | a | a | 9 |
| 49 | Polovina 2018 | a | a | a | a | Yes | Yes | a | a | b | 9 |
