# Supplementary material for: Impact of Metabolic Syndrome and It's Components on Prognosis in Patients With Cardiovascular Diseases: A Meta-Analysis
Source: Front Cardiovasc Med. 2021 Jul 15;8:704145. doi: 10.3389/fcvm.2021.704145 (PMC8319572; doi:10.3389/fcvm.2021.704145)
Supplement: Supplementary file 3 [file Table_3.docx]

**Table S3 Criteria for** **Clinical Diagnosis of Metabolic Syndrome**

| **clinical measure** | **NCEP-ATP III**  **（2001）[9]** | **NCEP-ATP III**  **（2005）[13]** | **IDF（2005）[10]** |
| --- | --- | --- | --- |
| Eligibility | any 3 of the following 5 features | any 3 of the following 5 features | Increased WC (population specific) plus any 2 of the following 4 features |
| Increased WC | WC≥102 cm (40 inches) in men, ≥88 cm (35 inches) in women | WC≥102 cm (40 inches) in men, ≥88 cm (35 inches) in women | ethnicity specific |
| Elevated triglycerides | ≥ 150 mg/dL (1.7 mmol/L) | ≥150 mg/dL (1.7 mmol/L) or on drug treatment for elevated triglycerides | TG≥150 mg/dL (1.7 mmol/L) or on TG Rx |
| Reduced HDL-C | ＜40 mg/dL (1.03 mmol/L) in men, ＜50 mg/dL (1.3 mmol/L) in women | ＜40 mg/dL (1.03 mmol/L) in men, ＜50 mg/dL (1.3 mmol/L) in women or on drug treatment for reduced HDL-C | HDL-C＜40 mg/dL (1.03 mmol/L) in men or ＜50 mg/dL (1.3 mmol/L) in women or on HDL-C Rx |
| Blood pressure | ≥130/85 mm Hg | ≥130 mm Hg systolic blood pressure or ≥85 mm Hg diastolic blood pressure or on antihypertensive drug treatment in a patient with a history of hypertension | ≥130 mm Hg systolic or ≥85 mm Hg diastolic or on hypertension Rx |
| Elevated fasting glucose | ＞110 mg/dL (includes diabetes) | ≥100 mg/dL or On drug treatment for elevated glucose | ≥100 mg/dL (includes diabetes) |
